# 

**Published:** 1904-05-15

**Authors:** 


THE
### DENTAL REGISTER

EDITED BY
### NELVILLE S. HOFF, D. D. S.,
ANN ARBOR, MICH.

Vol. LVIII. MAY 15, 1904. No. 5.

PRICE, ONE DOLLAR A YEAR IN ADVANCE.

PUBLISHED MONTHLY BY

SAMUEL A. CROCKER & CO.,

THE OHIO DENTAL AND SURGICAL DEPOT.

35 to 39 W. 5th St., Cincinnati, O.

Entered at the Postoffice at Cincinnati, O., as second-class mail matter.

